# HWJMSC-EVs promote cartilage regeneration and repair via the ITGB1/TGF-β/Smad2/3 axis mediated by microfractures

**DOI:** 10.1186/s12951-024-02451-2

**Published:** 2024-04-12

**Authors:** Zhian Chen, Tianhua Zhou, Huan Luo, Zhen Wang, Qiang Wang, Rongmao Shi, Zian Li, Rongqing Pang, Hongbo Tan

**Affiliations:** 1 Graduate School, Kunming Medical University, Kunming, Yunnan China; 2 Department of Orthopaedics, People’s Liberation Army Joint Logistic Support Force 920th Hospital, Kunming, Yunnan China; 3 Basic Medical Laboratory, People’s Liberation Army Joint Logistic Support Force 920th Hospital, Kunming, Yunnan China

**Keywords:** Chondrocytes, Regeneration, Microfractures, Human Wharton’s jelly MSCs, Extracellular vesicles

## Abstract

The current first-line treatment for repairing cartilage defects in clinical practice is the creation of microfractures (MF) to stimulate the release of mesenchymal stem cells (MSCs); however, this method has many limitations. Recent studies have found that MSC-derived extracellular vesicles (MSC-EVs) play an important role in tissue regeneration. This study aimed to verify whether MSC-EVs promote cartilage damage repair mediated by MFs and to explore the repair mechanisms. In vitro experiments showed that human umbilical cord Wharton’s jelly MSC-EVs (hWJMSC-EVs) promoted the vitality of chondrocytes and the proliferation and differentiation ability of bone marrow-derived MSCs. This was mainly because hWJMSC-EVs carry integrin beta-1 (ITGB1), and cartilage and bone marrow-derived MSCs overexpress ITGB1 after absorbing EVs, thereby activating the transforming growth factor-β/Smad2/3 axis. In a rabbit knee joint model of osteochondral defect repair, the injection of different concentrations of hWJMSC-EVs into the joint cavity showed that a concentration of 50 µg/ml significantly improved the formation of transparent cartilage after MF surgery. Extraction of regenerated cartilage revealed that the changes in ITGB1, transforming growth factor-β, and Smad2/3 were directly proportional to the repair of regenerated cartilage. In summary, this study showed that hWJMSC-EVs promoted cartilage repair after MF surgery.

**Graphical abstract:**

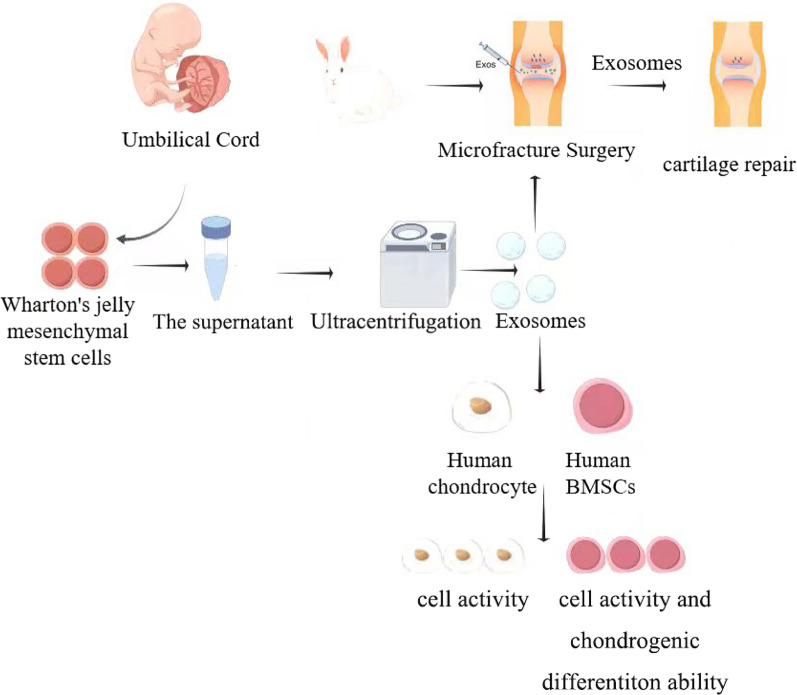

**Supplementary Information:**

The online version contains supplementary material available at 10.1186/s12951-024-02451-2.

## Introduction

There are currently approximately 240 million people worldwide with osteoarthritis [1]. Up to 12% of patients develop knee osteoarthritis after trauma [2], mainly because of the poor self-healing ability of cartilage and limitations in surgical techniques [3–5]. The microfracture (MF) technique is used as a first-line treatment for full-thickness cartilage injuries with a diameter of less than 4 cm in clinical practice, this MF technique repairs cartilage injuries by stimulating the release of bone marrow-derived mesenchymal stem cells (BMSCs), the BMSCs form fibrocartilage, which alleviates the patient’s symptoms but has reduced mechanical properties compared with normal articular cartilage [6, 7]. Recent tissue engineering strategies that induce BMSCs to differentiate into cartilage and promote chondrocyte regeneration are considered a promising method for joint cartilage regeneration [8, 9]. However, there is controversy regarding the induction of BMSCs to differentiate into cartilage and promote chondrocyte regeneration at the RNA and protein levels [10]. The mechanism of inducing differentiation of BMSCs has a significant effect on the function of stem cells after differentiation, which is of great significance in tissue engineering.

Extracellular vesicles (EVs) are released by endocytosis in all cells, prokaryotes, and eukaryotes. Depending on their source, EVs contain different DNA, RNA, lipids, metabolites, and cytosolic and cell surface proteins [11], and can undergo intercellular signaling and alter the biological activity of recipient cells through their own substances [12, 13]. Based on extensive research [14, 15], previous experiments have shown that human umbilical cord Wharton’s jelly mesenchymal stem cell (hWJMSC)-derived EVs (hWJMSC-EVs) have significant effects in inhibiting chondrocyte apoptosis, improving chondrocyte viability, and promoting cell cycle progression [16]. Furthermore, mesenchymal stem cell (MSC)-derived EVs (MSC-EVs) achieve tissue regeneration by regulating transforming growth factor (TGF)-β [17] in spinal cord injury [18], kidney disease [19], osteochondral injury, and osteoarthritis. The activation and release of TGF-β have the function of recruiting MSCs and inducing MSC differentiation, thereby enabling MSCs to repair damaged tissues [20, 21]. However, the molecular mechanism by which MSC-EVs activate TGF-β in cartilage regeneration is not fully understood.

Proteomic analysis of hWJMSC-EVs by mass spectrometry has shown that hWJMSC-EVs are rich in various proteins [22]. Among these proteins, integrin beta-1 (ITGB1) has aroused research interest. As the most common and important receptor of type 2 collagen (COL-II), ITGB1 not only connects chondrocytes and the extracellular matrix (ECM), but also plays a crucial role in signal transduction [23–25]. Studies have suggested that ITGB1 binds to the N-terminal fragment of TGF-β to activate TGF-β [26]. However, the mechanisms of both ITGB1 and TGF-β in cartilage regeneration and the induction of MSC differentiation are not yet understood. The TGF-β/Smad superfamily plays an indispensable role in the biological process of cartilage regeneration and has multiple branches, among which the TGF-β/Smad2/3 signaling pathway promotes chondrogenesis and inhibits chondrocyte hypertrophy [27–29]. Studies have shown that a lack of TGF-β can lead to a significant decrease in ECM, and the Smad2/3 signaling pathway is significantly downregulated in the later stages of cartilage injury [30]. The TGF-β signaling pathway is regulated by BMSCs, microglia, and cancer-related EVs [31–33], while ITGB1 is a stably expressed molecule in MSCs [23]. Therefore, hWJMSC-EVs may promote chondrogenic differentiation and regeneration of BMSCs by regulating the TGF-β/Smad2/3 axis through the regulation of ITGB1.

**Fig. 1:**
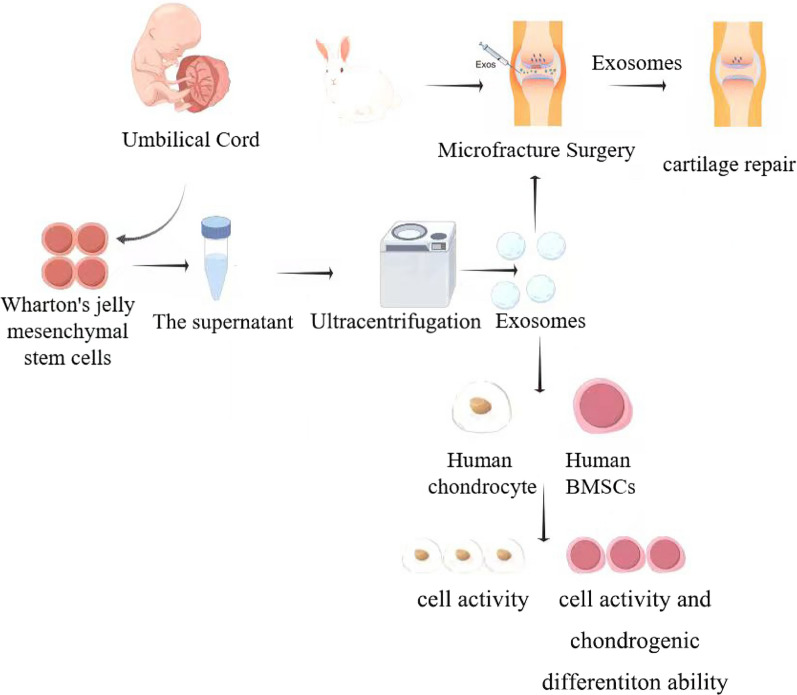
Schematic illustration of the whole study. Exosomes were extracted from Wharton’s jelly mesenchymal stem cells by ultracentrifugation. Animal experiments were conducted by injecting exosomes through the joint cavity into rabbits with cartilage damage to evaluate the ability of exosomes to repair cartilage damage in rabbit knee joints. Exosomes were co-cultured with human bone marrow-derived mesenchymal stem cells (hBMSCs) and chondrocytes in cell experiments to detect the chondrogenic differentiation ability of hBMSCs and the proliferative ability of chondrocytes

To explore the differentiation of BMSCs into transparent chondrocytes after MF stimulation, we first combined hWJMSC-EVs with MF-stimulated BMSCs to repair cartilage defects. Then, from the perspective of inducing stem cell differentiation and cartilage regeneration, we preliminarily examined the mechanism by which EVs affect BMSCs and chondrocytes (Fig. 1).

## Materials and methods

### Cell culture and treatment

Human BMSCs (hBMSCs; 5 × 10^5^ cells per T25 flask) and human chondrocytes (5 × 10^5^ cells per T25 flask) were obtained from Procell (Wuhan, China), while hWJMSCs were purchased from Yinfeng Biotechnology (Kunming, China). The cells were placed into two tubes: one tube for passage and the other for cryopreservation. All cells were cultured with DMEM (Gibco, Logan City, UT, USA) containing 10% fetal bovine serum (BIOIND) in a humidified atmosphere with 5% CO_2_ at 37 °C. All cells were consistently used at passages 3 to 5 for subsequent experiments.

Chondrogenic induction medium (Pricella, Wuhan, China) was used to induce the differentiation of hBMSCs and chondrocytes. After 21 days, the ability of BMSCs to differentiate into chondrocytes was assessed using Alcian Blue staining.

### Cell viability assay

The viability of hBMSCs and chondrocytes was determined using the Cell Counting Kit-8 (CCK-8, Sangon Biotech, Shanghai, China). Cells were seeded in 96-well plates in 5% CO_2_ at 37 °C; CCK-8 solution (10 μL) was added to each well and cultivated for 1 h. Next, a microplate reader (BioTek Instruments Inc.) was used to measure the absorbance at 450 nm.

### Alcian Blue staining

The medium was aspirated from the chondrocytes and hBMSCs, washed with DPBS (BI; 02–023-1, 0.2 ml/well), and fixed in 4% paraformaldehyde at room temperature for 15–30 min. We then dissolved 0.2 g of Alcian Blue 8GX (Sigma; A-3157) in 20 ml of acidified isopropanol (0.1% HCl in isopropanol) to prepare a 1% Alcian Blue solution. The 1% Alcian Blue solution (0.2 ml) was added to each well containing cells, and the plates were maintained at room temperature overnight in the dark. The dye solution was aspirated and removed, and the cells were washed two to three times with 0.2 ml of 0.1 N HCl. An inverted light microscope was used to photograph images.

### Isolation of HWJMSC-EVs

HWJMSC-EVs were isolated as described previously [16]. Ribo™ Exosome Isolation Reagent (Ribobio, China) was used to isolate EVs. Briefly, the hWJMSCs cell culture medium was placed in a 15-ml centrifugal tube and centrifuged at room temperature at 2000×*g* for 30 min. A 15-ml centrifuge tube containing 2 ml of Ribo™ Exosome Isolation Reagent was then filled with the centrifuged supernatant and left overnight. Next, these mixtures were centrifuged at 1500×*g* for 30 min at 4 °C so that the EVs were contained in the pellet at the bottom of the centrifugal tube. The final pellets of exosomes were suspended in PBS and stored at − 80 °C. The Bradford method (Bio-Rad, Hercules, USA) was used to quantify the protein concentration of the exosomes.

### Immunofluorescence staining

PKH67 immunofluorescence staining was performed as described previously [16]. Briefly, hWJMSC-EV-treated hBMSCs and chondrocytes were fixed in 4% paraformaldehyde for 15 min and permeabilized. Then, the cells were incubated overnight at 4 °C with PKH67 (dilution 1:200; cat. no. ab204951; Abcam). After 12 h, the cells were incubated with secondary antibody for 2 h at room temperature. A Nikon Eclipse 80i microscope (Nikon Corporation) was used to observe the staining results.

### Transmission electron microscopy

The exosome samples were dropped onto a copper grid coated with polyformaldehyde carbon, dried for 30 min, and fixed in 2% paraformaldehyde. Next, the grids were stained with 2% uranyl acetate and observed using a transmission electron microscope.

### Western blot analysis

The cells (hWJMSCs, hBMSCs, and chondrocytes) and tissues were extracted used the Total Protein Extraction Kit (Sangon Biotech). Next, BCA Protein Assay kits (Sangon Biotech) were used to determine the protein concentration. The total protein was separated with 10% sodium dodecyl sulfate–polyacrylamide gel electrophoresis, transferred to P membrane, and blocked in 5% skimmed milk. Subsequently, the membrane was incubated overnight with exosome-associated primary antibodies (CD63, CD9, and TSP70) as well as the following primary antibodies: ITGB1, TGF-β, p-Smad2/3, Smad2/3, Smad6, COLL-II, and β-actin. The next day, all membranes were washed with PBST three times, and then incubated with the appropriate secondary antibody for 1 h. An enhanced chemiluminescence reagent (Sangon Biotech) was used to visualize the immunoreactive bands. The strength of each band was detected using ImageJ software (National Institutes of Health, USA).

### Animal model and treatment

Sixty adult New Zealand white rabbits were anesthetized with pentobarbital sodium (3%, 1 ml/kg; Shandong Huamu Pharmaceutical Co., Ltd.). A ring drill was used to create a bone cartilage defect (diameter 5 mm, depth 2 mm) in the trochlear groove of the rabbits [6]. Immediately after the bone cartilage defect was created, a drill bit with a diameter of 0.7 mm was used to drill five 2-mm-deep holes at intervals of 0.9 mm (i.e., to create MFs) [34]. The surgical site was then sutured closed with 3–0 Vicryl® (Aixikang Co., Ltd.). After suturing, 48 rabbits were randomly divided into four groups (n = 12 rabbits in each group) that were injected with 1 ml of HWJMSC-EVs at concentrations of 5, 25, 50, and 100 µg/ml, respectively, into the joint cavity by a veterinarian [14]. The concentration of the injected HWJMSC-Evs was marked on the cage of each rabbit, and the labeled concentration was injected into the knee joint again on the 3rd and 7th days after surgery. Veterinary staff from the animal research laboratory of a tertiary hospital provided standard postoperative care for the rabbits, including infection prevention, analgesia, wound observation, and disinfection. This study was approved by the Ethics Committee of the 920th Hospital of the Joint Logistics Support Force of the People’s Liberation Army (Ethics 2022-072-01).

### Gross observations

Six rabbits in each group (6 knees in each group) were randomly selected and euthanized by intravenous pentobarbital injection (3%, 100–150 mg/kg, Shandong Huamu Pharmaceutical Co., Ltd.) at 3 and 6 months after surgery. Immediately after euthanasia, the distal femur was dissected. Three observers who were blinded to the animal groupings photographed the femurs, performed gross observations, and evaluated the cartilage using the International Society for Cartilage Repair (ICRS) scoring system [6].

### Micro-CT scanning

After fixing the knee joint in neutral buffered formalin for 1 week, the distal femur was wrapped in paraffin film to prevent dryness during scanning. The knee joints were scanned, the images were reconstructed using Nrecon software (Bruker microCT, Kontech, Belgium), and the same threshold (cross-sectional image) of each femur was selected to evaluate subchondral bone healing.

### Histology assessment

The samples were fixed, decalcified, dehydrated, and embedded in paraffin. The paraffin-embedded samples were sliced at 4 µm and dewaxed to water. Staining was performed using a hematoxylin and eosin (H&E) staining kit (G1120, Solarbio, Shanghai, China), Alcian Blue staining kit (G1560, Solarbio), and Safranin O staining kit (G1371, Solarbio). Positive fluorescence microscopy (NIKON ECLIPSE E100, Nikon) was used and panoramic scanning (NIKON DS-U3, Nikon) was performed using an imaging system. Three researchers from relevant fields blindly scored the stained sections using the tissue morphology scoring system [35] to evaluate the cartilage and subchondral bone repair.

### Immunohistochemistry

The paraffin-embedded samples were sectioned, dewaxed to water, and underwent antigen extraction. After incubating the samples at room temperature for 30 min with 3% hydrogen peroxide in the dark, 10% goat serum (K5007, Dako, Shanghai, China) was added and the samples were maintained at 25 °C for 1 h. The samples were then incubated overnight at 4 °C with antibodies to COLL-I (14695-1-AP, San Ying Biotechnology, Wuhan, China), COLL-II (MA1-37493, Thermo Fisher Scientific, USA), and COLL-III (22734-1-AP, San Ying Biotechnology) at appropriate dilutions. On the second day, the samples were added to a 37 °C constant temperature incubator with enhanced chemical reaction solution for 20 min and washed with PBS before goat anti-rabbit secondary antibody (K5007, Dako) was added to cover the labeled tissue. The samples were incubated at room temperature for 50 min. The color was developed by mixing with diaminobenzidine solution (DA1016, Solarbio) at room temperature for 5–10 min before being washed with distilled water, re-stained, dehydrated, and sealed. Positive fluorescence microscopy (NIKON ECLIPSE E100, Nikon) and panoramic scanning (NIKON DS-U3, Nikon) were performed using an imaging system.

### Statistical analysis

All experiments were repeated three times independently. GraphPad Prism 8 (GraphPad Software, Inc.) was used to statistically analyze and graph all data. The data were showed as mean ± standard deviation (SD). The Student’s t test or one-way ANOVA were used for comparisons among groups. *p* < 0.05 was considered to indicate a statistically significant difference.

## Results

### HWJMSCs and hWJMSC-EVs promoted the activity of hBMSCs and chondrocytes

**Fig. 2:**
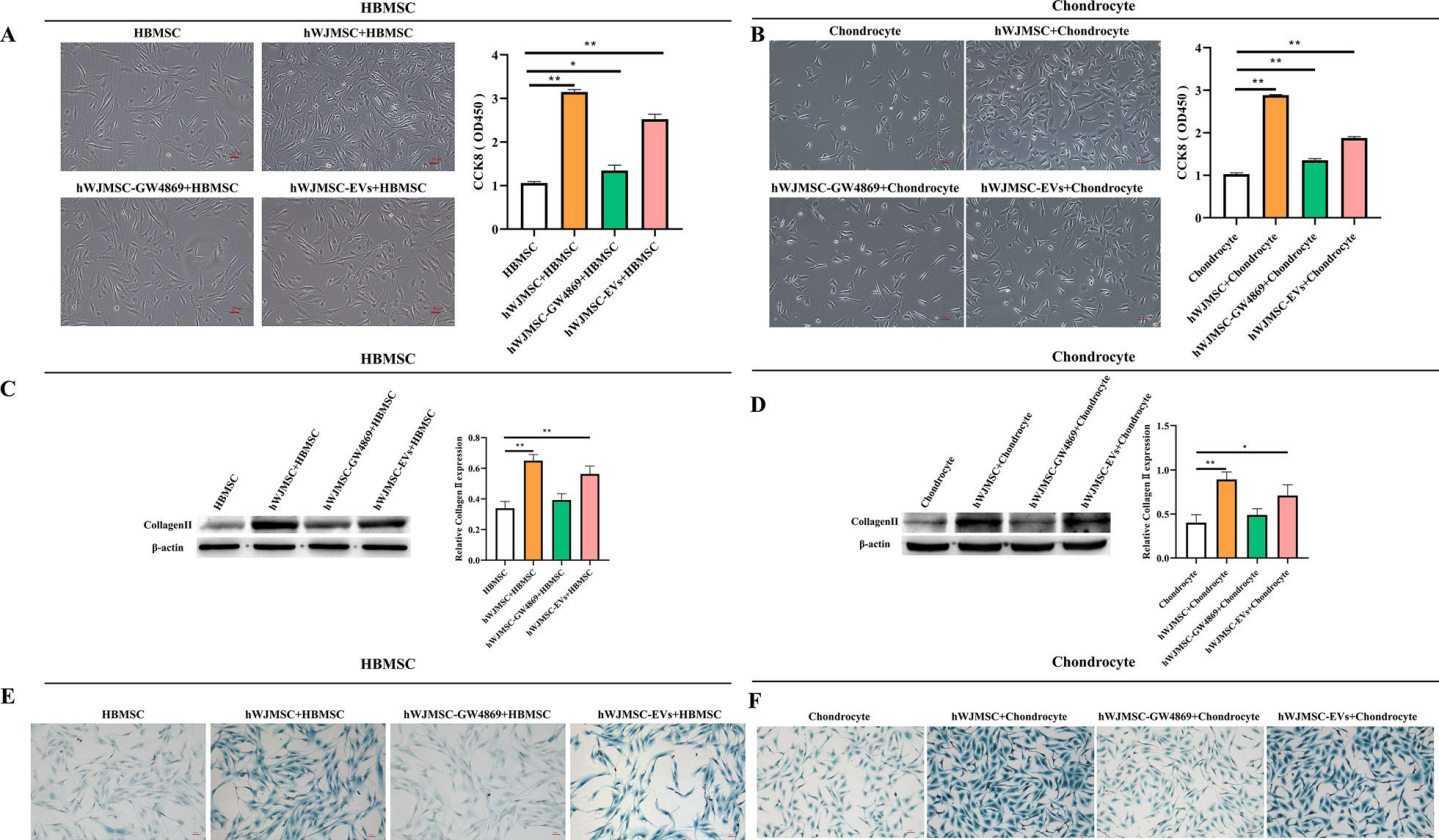
HWJMSCs and HWJMSC-EVs promoted the activity of hBMSCs and chondrocytes. **A**, **B** The viability of cells treated with hWJMSCs and HWJMSC-EVs was detected by CCK8 assay and observed under light microscopy. Scale bar = 50 µm. Mean ± SD, n = 3. *P < 0.05, **P < 0.01. **C**, **D** The expression of COL-II in cells (hBMSCs and chondrocytes) was detected by Western blotting. Mean ± SD, n = 3. *P < 0.05, **P < 0.01. **E**, **F** Alcian Blue staining of cells treated with hWJMSCs and HWJMSC-EVs. Scale bar = 50 µm. Mean ± SD, n = 3

As shown in Fig. 2A and B, co-culture with hWJMSCs promoted the viability of hBMSCs and chondrocytes, but resulted in no differences in cellular morphology. Because our previous study found that hWJMSCs-EVs alleviated IL-1β-induced chondrocyte damage [16], the hWJMSCs-EVs were isolated to investigate the effect of hWJMSCs-EVs on the viability of hBMSCs and chondrocytes. The results showed that hWJMSCs-EVs promoted the vitality of hBMSCs and chondrocytes, but the phenomenon was suppressed by an exosome inhibitor (GW4869; 10 μm) (Fig. 2A, B). After discovering that hWJMSCs and hWJMSCs-EVs promoted the proliferation of hBMSCs and chondrocytes, we speculated that hWJMSCs and hWJMSCs-EVs might promote the chondrogenic differentiation ability of hBMSCs. Therefore, hBMSCs co-cultured with hWJMSCs or treated with hWJMSCs-EVs were cultured in a chondrogenic medium for 14 days. Alcian Blue staining results showed that hWJMSCs and hWJMSCs-EVs promoted glycosaminoglycan staining of hBMSCs. Similarly, normal chondrocytes co-cultured with hWJMSCs or treated with hWJMSCs-EVs also showed significantly increased glycosaminoglycan staining (Fig. 2E, F). Subsequently, the proteins of hBMSCs and chondrocytes after chondrogenic induction were extracted, and the expression of COL-II was detected. The results showed that COL-II was significantly upregulated in the hWJMSC and hWJMSC-EV groups (Fig. 2C, D).

### EVs from hWJMSCs were isolated and taken up by BMSCs and chondrocytes

**Fig. 3:**
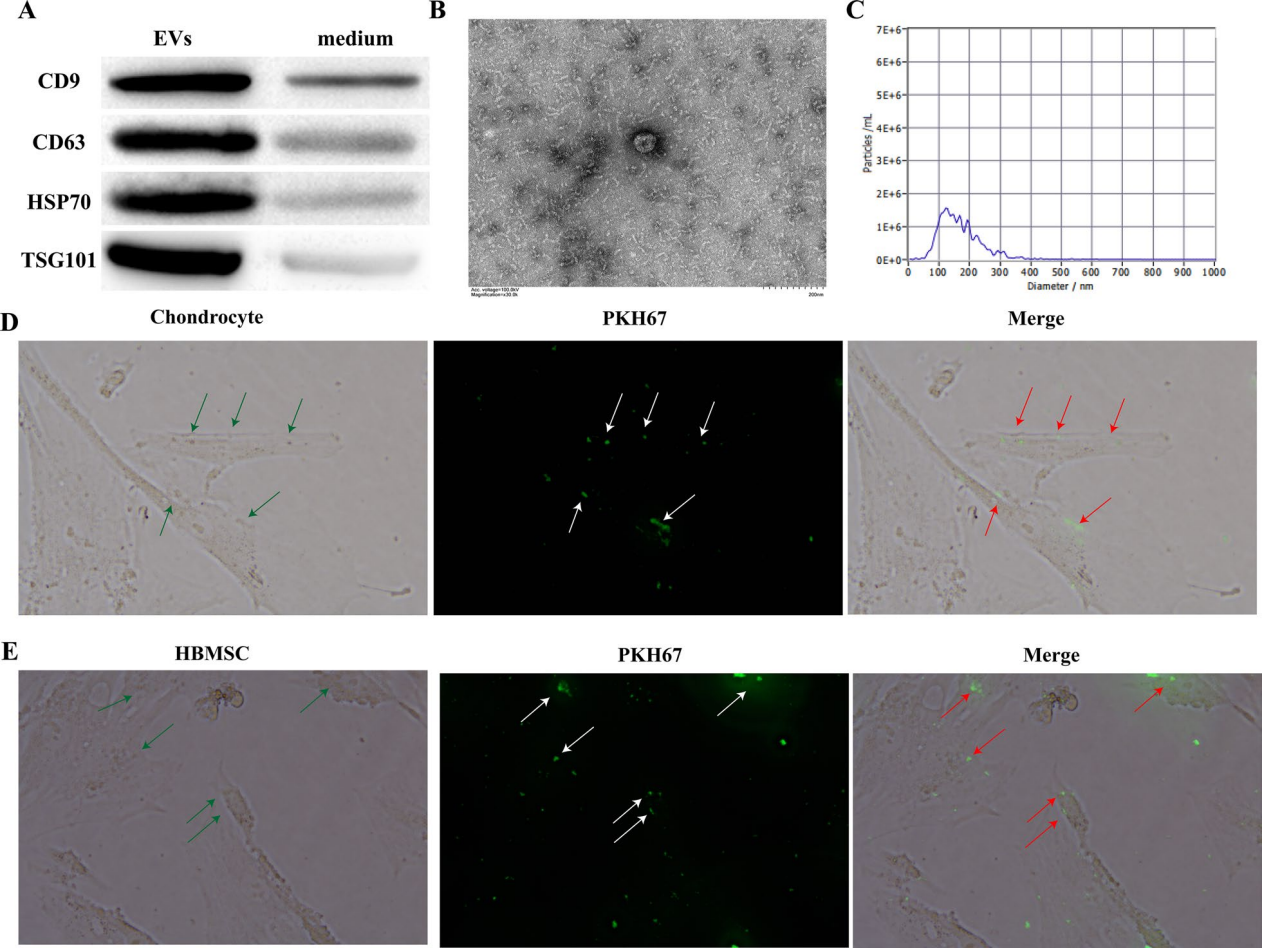
Isolation of EVs and characterization of hWJMSCs. **A** Western blot analysis of the exosomal markers CD9,CD63, TSG101 and TSP70. **B** Transmission electron microscopic images of hWJMSC-EVs. Scale bar = 200 nm. **C** Nanoparticle tracking analysis of hWJMSC-EVs. **D**, **E** Fluorescence images of chondrocytes and HBMSCs incubated with PKH67-labeled hWJMSC-EVs (green). Green arrow indicates chondrocytes; white arrow indicates hWJMSC-EVs; red arrow shows HWJMSC-EVs entering chondrocytes. Scale bar = 100 µm

Western blotting showed that the EV marker proteins CD9, CD63, TSG101 and TSP70 were highly expressed in the isolated EVs (Fig. 3A). Furthermore, electron microscopy showed that the EVs had a complete structure in a round shape (Fig. 3B). Nanoparticle tracking analysis showed that the size range was 60–350 nm, which met the EV size requirements (Fig. 3C). PKH67 fluorescence detection of the location of EVs in cells showed that PKH67 was localized in the cytoplasm of BMSCs and chondrocytes (Fig. 3D, E). These results indicated that HWJMSC-EVs were taken up by hBMSCs and chondrocytes.

### HWJMSCs and HWJMSC-EVs upregulated ITGB1 and TGF-β in hBMSCs and chondrocytes

**Fig. 4:**
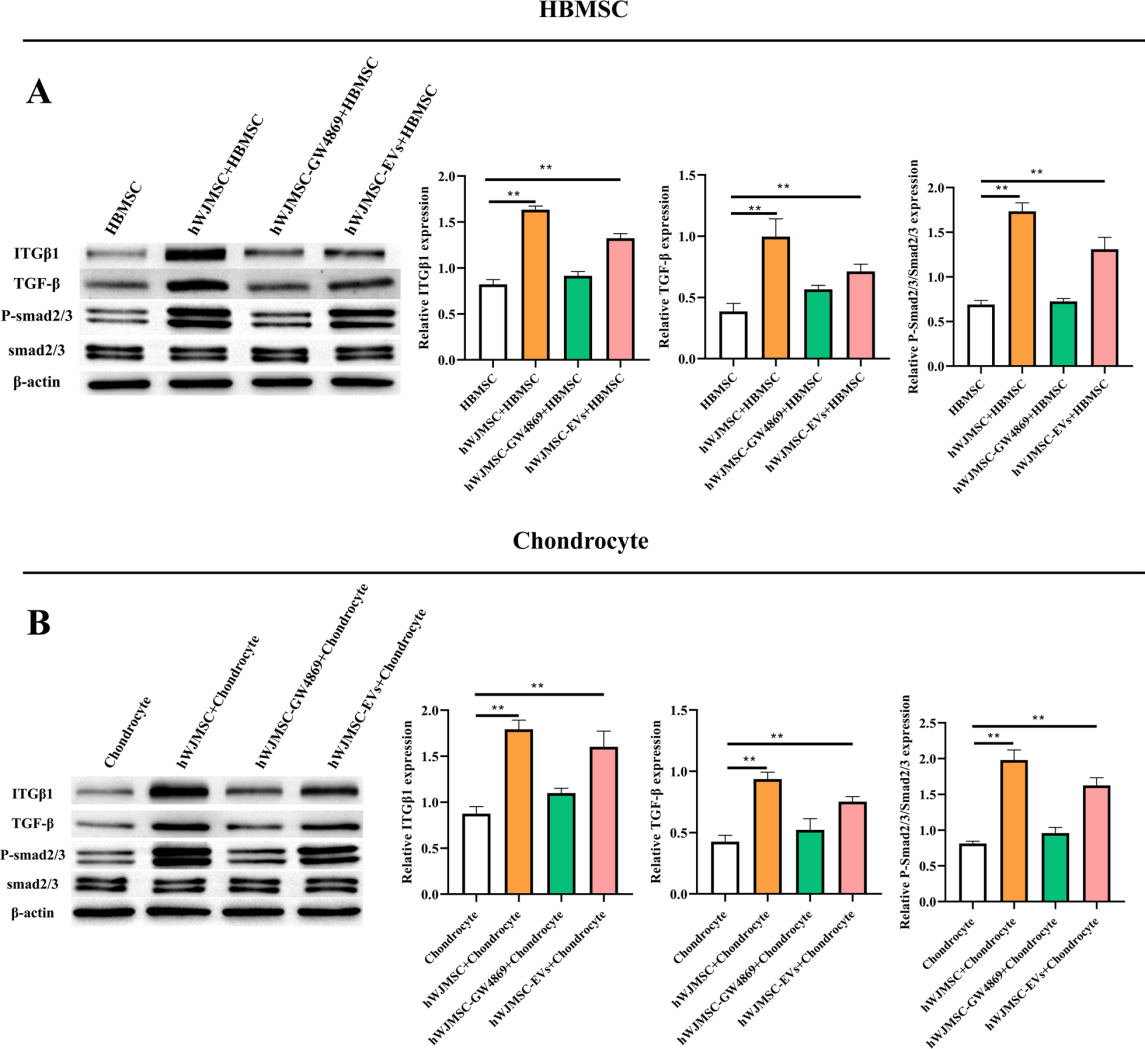
HWJMSCs and HWJMSC-EVs upregulated ITGB1 and TGF-β in hBMSCs and chondrocytes. The expressions of ITGB1, TGF-β, p-Smad2/3, and Smad2/3 in hBMSCs and chondrocytes detected by Western blotting. Mean ± SD, n = 3. *P < 0.05, **P < 0.01

ITGB1 may be involved in the regulatory effects of hWJMSCs and hWJMSC-EVs on hBMSCs and chondrocytes. ITGB1 was highly expressed in hWJMSC-EVs, while ITGB1 was highly expressed in hBMSCs and chondrocytes co-cultured with hWJMSCs or treated with hWJMSC-EVs (Fig. 4A, B). Furthermore, TGF-β was highly expressed in hBMSCs and chondrocytes co-cultured with hWJMSCs or treated with hWJMSC-EVs. TGF-β also upregulated the phosphorylation level of Smad2/3 but did not affect the expression of Smad6 (Fig. 4A, 4B; Additional file 1: Fig. S1).

### HWJMSCs and HWJMSC-EVs promoted the expression of ITGB1 in BMSCs and chondrocytes

**Fig. 5:**
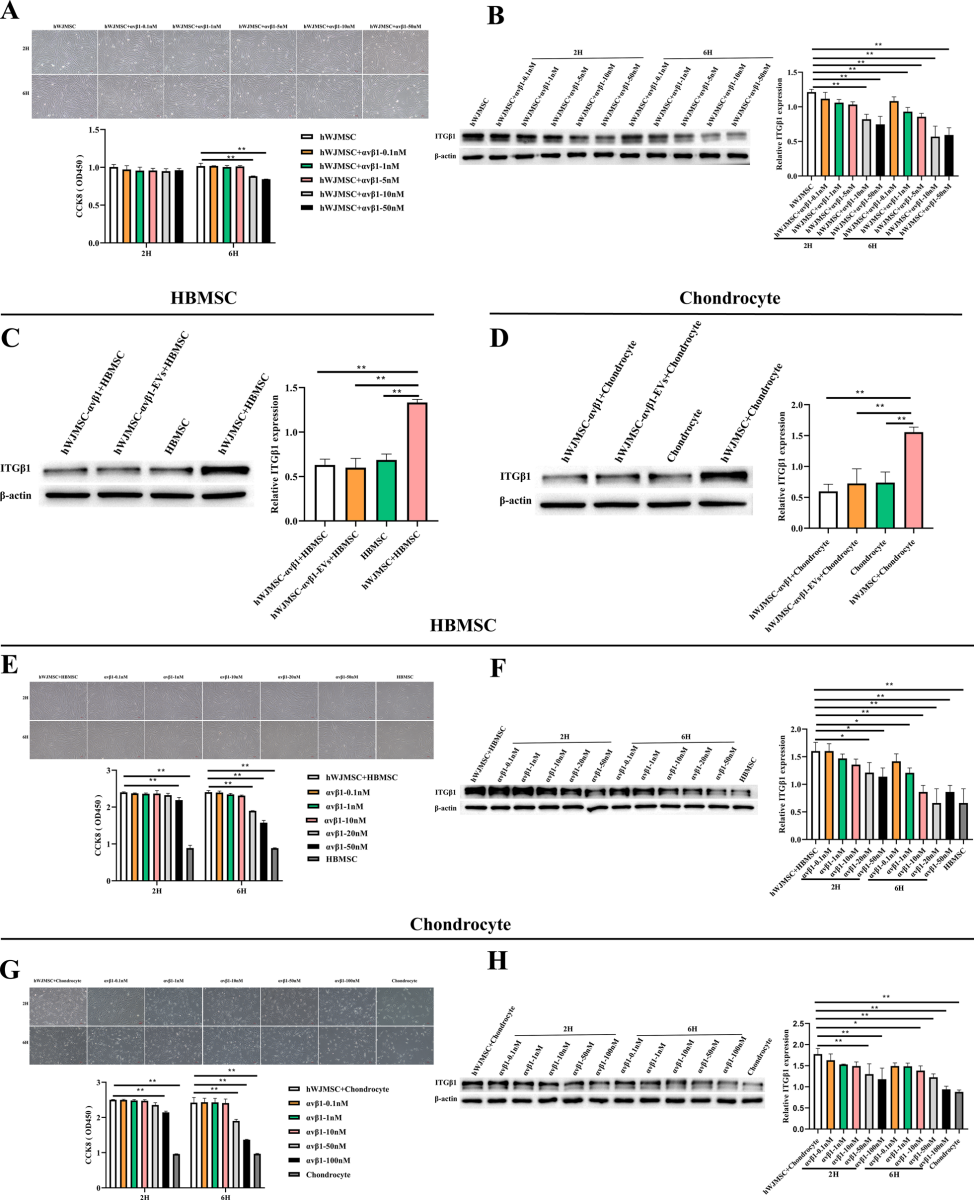
HWJMSCs and HWJMSC-EVs promoted the expression of ITGB1 in BMSCs and chondrocytes. **A**, **E**, **G** The viability of treated hWJMSCs, hBMSCs, and chondrocytes was detected by CCK8 assay and observed under light microscopy. Scale bar = 50 µm. Mean ± SD, n = 3. *P < 0.05, **P < 0.01. **B**–**D**, **F**, **H** The expressions of ITGB1 in cells (hWJMSCs, hBMSCs and chondrocytes) detected by Western blotting. Mean ± SD, n = 3. *P < 0.05, **P < 0.01

HWJMSCs were treated with 0.1, 1, 5, 10, and 50 nM of an ITGB1 inhibitor (αvβ1) for 2 h and 6 h, respectively. Treatment with 10 nm of αvβ1 for 6 h significantly inhibited the expression of ITGB1 without affecting cell viability (Fig. 5A, B). Treatment of hWJMSCs and hWJMSC-EVs with αvβ1 significantly downregulated the levels of ITGB1 in hBMSCs and chondrocytes (Fig. 5C, D). In addition, the expression of ITGB1 was downregulated by the treatment of hBMSCs with 50 nm of αvβ1 for 6 h and by the treatment of chondrocytes with 100 nm of αvβ1 for 6 h (Fig. 5E–H; Additional file 1: Fig. S2).

### HWJMSCs and HWJMSC-EVs promoted the activity of BMSCs and chondrocytes by regulating the ITGB1/TGF-β axis

**Fig. 6:**
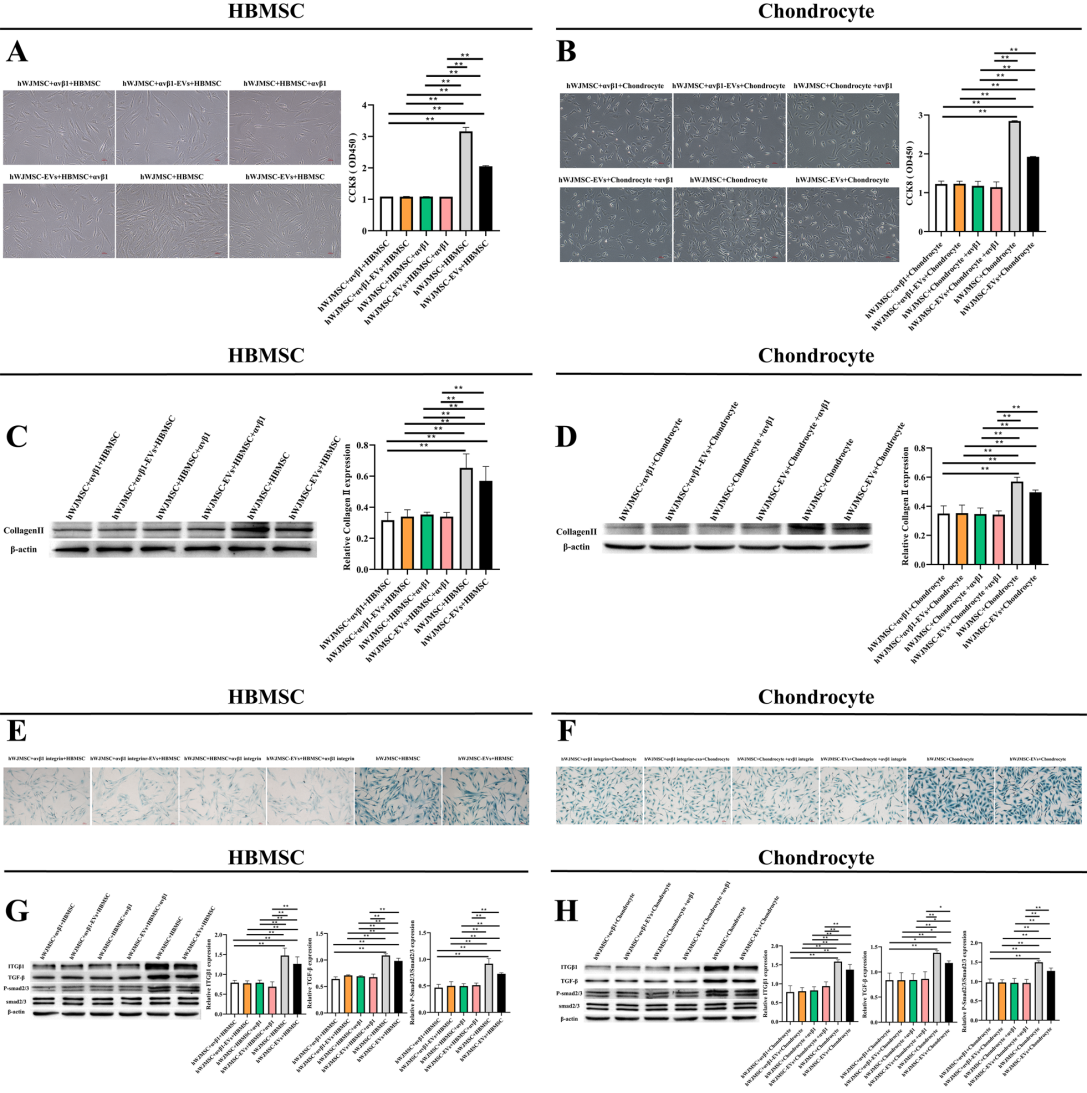
HWJMSCs and HWJMSC-EVs promoted the activity of BMSCs and chondrocytes by regulating the ITGB1/TGF-β axis. **A**, **B** The viability of treated cells detected by CCK8 assay and observed under light microscopy. Scale bar = 50 µm. Mean ± SD, n = 3. *P < 0.05, **P < 0.01. **C**, **D**, **G**, **H** The expressions of COL-II, ITGB1, TGF-β, p-Smad2/3, and Smad2/3 in hBMSCs and chondrocytes detected by Western blotting. Mean ± SD, n = 3. *P < 0.05, **P < 0.01. **E**, **F** Alcian Blue staining of hBMSCs and chondrocytes. Scale bar = 50 µm. Mean ± SD, n = 3

HBMSCs affected by αvβ1 were divided into six groups: HWJMSCs + αvβ1 + hBMSCs, hWJMSCs + αvβ1-EV + hBMSCs, hWJMSCs + hBMSCs + αvβ1, hWJMSC-EVs + hBMSCs + αvβ1, hWJMSCs + hBMSCs, and hWJMSC-EVs + hBMSCs. Chondrocytes affected by αvβ1 were divided into six groups: hWJMSCs + αvβ1 + chondrocytes, hWJMSCs + αvβ1-EV + chondrocytes, hWJMSCs + chondrocytes + αvβ1, hWJMSC-EVs + chondrocytes + αvβ1, hWJMSCs + chondrocytes, and hWJMSC-EVs + chondrocytes. The CCK8 results showed that αvβ1 inhibited the effect of hWJMSCs and hWJMSC-EVs in promoting the viability of hBMSCs and chondrocytes (Fig. 6A, B). The Alcian Blue staining results showed that αvβ1 downregulated the effect of hWJMSCs and hWJMSC-EVs in promoting the glycosaminoglycan production of hBMSCs and chondrocytes (Fig. 6E, F). Western blotting results showed that COL-II was downregulated in hBMSCs and chondrocytes affected by αvβ1 (Fig. 6C, D). In addition, αvβ1 downregulated the expressions of TGF-β and Smad2/3 in hBMSCs and chondrocytes (Fig. 6G, H).

### hWJMSC-EVs combined with BMSCs promoted articular cartilage regeneration

#### Gross observation and micro-CT evaluation of subchondral bone healing

**Fig. 7:**
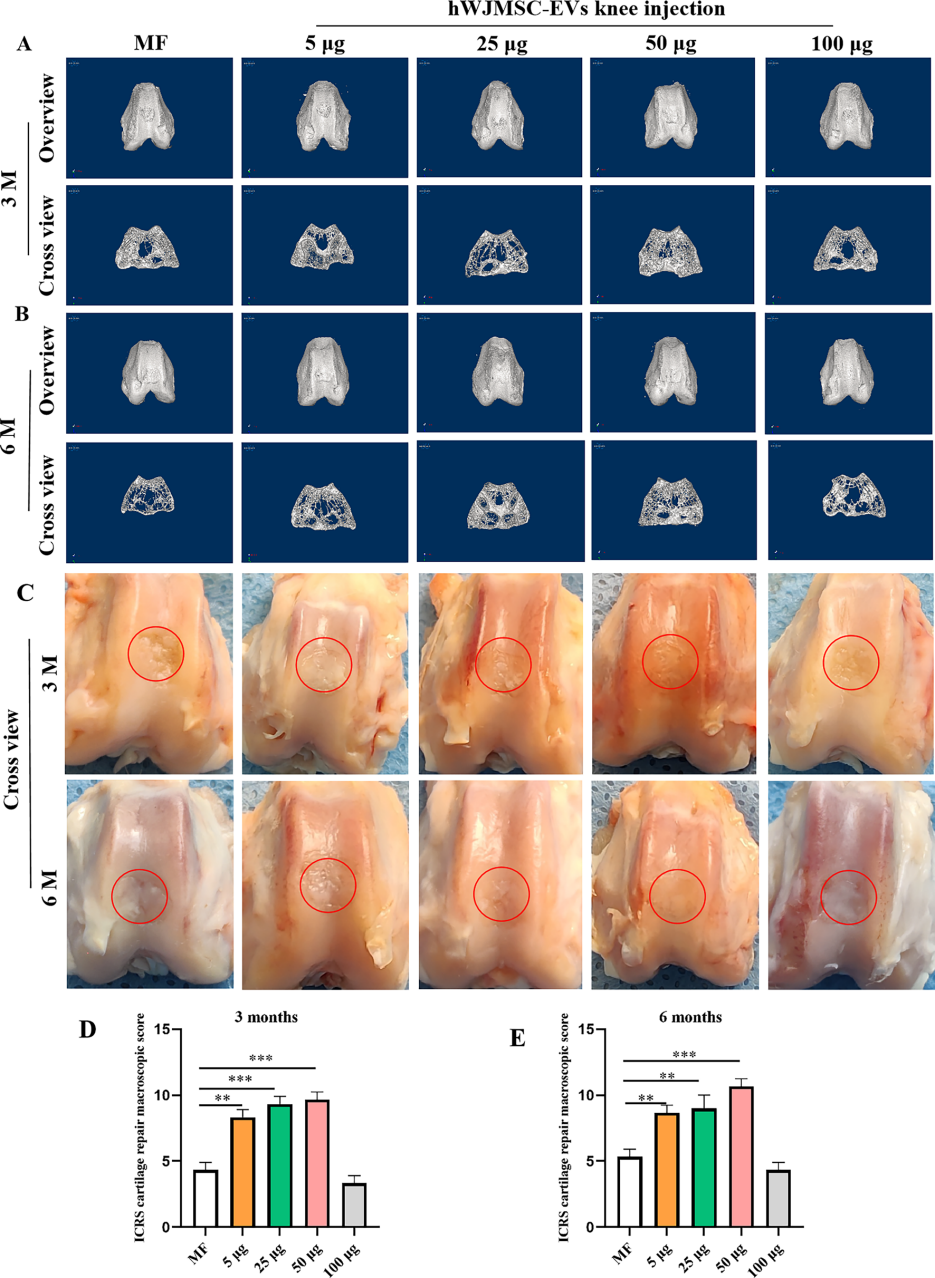
Gross observation and micro-CT images. **A**, **B** Micro-CT 3D images and cross-sectional view of the center of the osteochondral defect. **C**–**E** Gross image of the osteochondral defect healing and ICRS score. The red circles show the locations of the defect. Mean ± SD. *P < 0.05, **P < 0.01, **P < 0.005

Micro-CT scanning performed at 3 and 6 months postoperatively (Fig. 7A, B) showed that the subchondral bone defect area in the 50 µg/ml group was basically healed, while non-healing of the subchondral bone and incomplete healing of the MF site were observed in the MF group and the 5, 25, and 100 µg/ml groups (Fig. 7A). The cross-sectional view of the middle area of the defect further showed that the 50 µg/ml group had optimal subchondral bone healing. At 6 months postoperatively, reconstructed micro-CT images showed incomplete healing of the MF holes in the MF group and 100 µg/ml group, and complete healing in all other groups. Cross-sectional images showed that the bone trabeculae in the 50 µg/ml group had healed, while bone trabecular regeneration was observed in the MF group and the 5, 25, and 100 µg/ml groups. Among the non-healed groups, the bone trabecular regeneration was poorer in the MF and 100 µg/ml groups (Fig. 7B).

The macroscopic cartilage regeneration at 3 and 6 months postoperatively is shown in Fig. 7C. At 3 months postoperatively, the MF and 100 µg/ml groups had only a small amount of fibrous tissue-like structures with rough surfaces and significant local defects. The height of the repaired tissue at 3 months postoperatively in the 5 and 25 µg/ml groups was significantly lower than that of the surrounding normal cartilage, with uneven surfaces and clear defect boundaries. At 3 months postoperatively in the 50 µg/ml group, the repaired tissue had filled the defect area, but the surface was uneven. At 6 months postoperatively, the repaired tissue levels in the MF and 100 µg/ml groups were lower than the levels of the surrounding normal cartilage. The height of the repaired tissue at 6 months postoperatively in the 5 and 25 µg/ml groups was the same as that of the surrounding normal cartilage, but the surface was uneven. At 6 months postoperatively, the 50 µg/ml group had no obvious boundaries with the surrounding normal cartilage, and the surface of the regenerated cartilage was smooth (Fig. 7C). The ICRS macroscopic scores at 3 and 6 months showed that the cartilage repair was significantly better in the 50 µg/ml group than in the other groups (Fig. 7D, E).

#### Cartilage repair evaluated with H&E staging and subchondral bone scoring

**Fig. 8:**
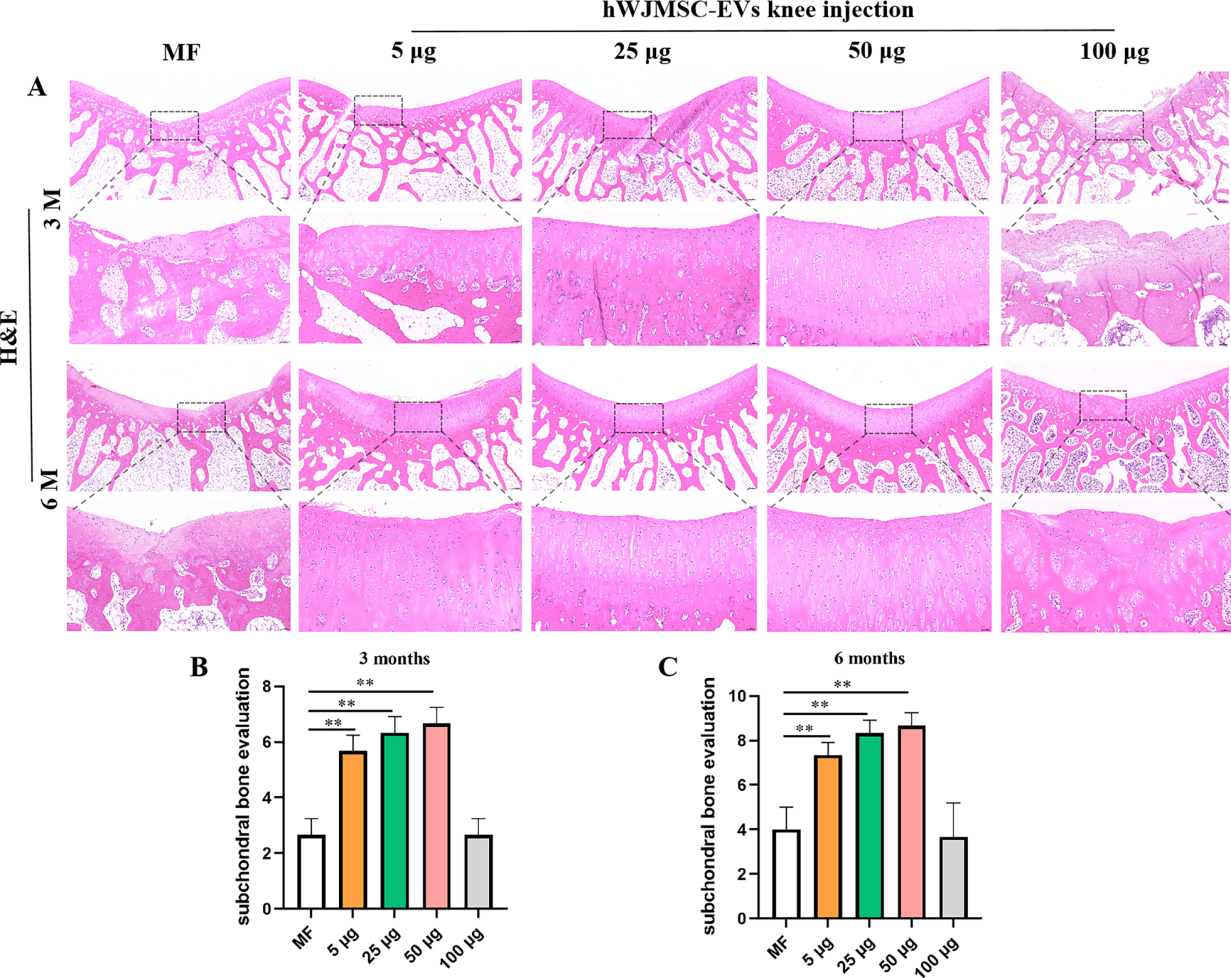
H&E staging and subchondral bone scoring. **A** H&E staging of regenerated cartilage. **B**, **C** Subchondral bone score, mean ± SD. Scale bar = 200 µm and 50 µm. *P < 0.05, **P < 0.01, **P < 0.005

The H&E staining results were similar to the macroscopic evaluation results (Fig. 8A). At 3 and 6 months postoperatively, the repaired tissue in the 50 µg/ml group had fully fused with the normal cartilage, and the subchondral bone reconstruction was good. At 3 months postoperatively, there was a small amount of regenerated cartilage in the MF and 100 µg/ml groups, with disordered and loose regenerated cartilage and fibrous tissue. At 6 months postoperatively, there was a small amount of regenerated cartilage presenting as flocculent fibrous tissue in the MF and 100 µg/ml groups. The subchondral bone had healed at 6 months postoperatively in the 5 and 25 µg/ml groups, and the surface of the regenerated cartilage had fused with the normal cartilage; a large amount of fibrous tissue was visible in the 5 µg/ml group, and the central crack of the regenerated cartilage was visible in the 25 µg/ml group. The subchondral bone score at 6 months postoperatively tended to be better in the 50 µg/ml group than the other groups, and was significantly better in the 50 µg/ml group compared with that in the MF group (Fig. 8B, C).

#### Evaluation of cartilage repair with Alcian Blue staining and Safranin O staining

**Fig. 9:**
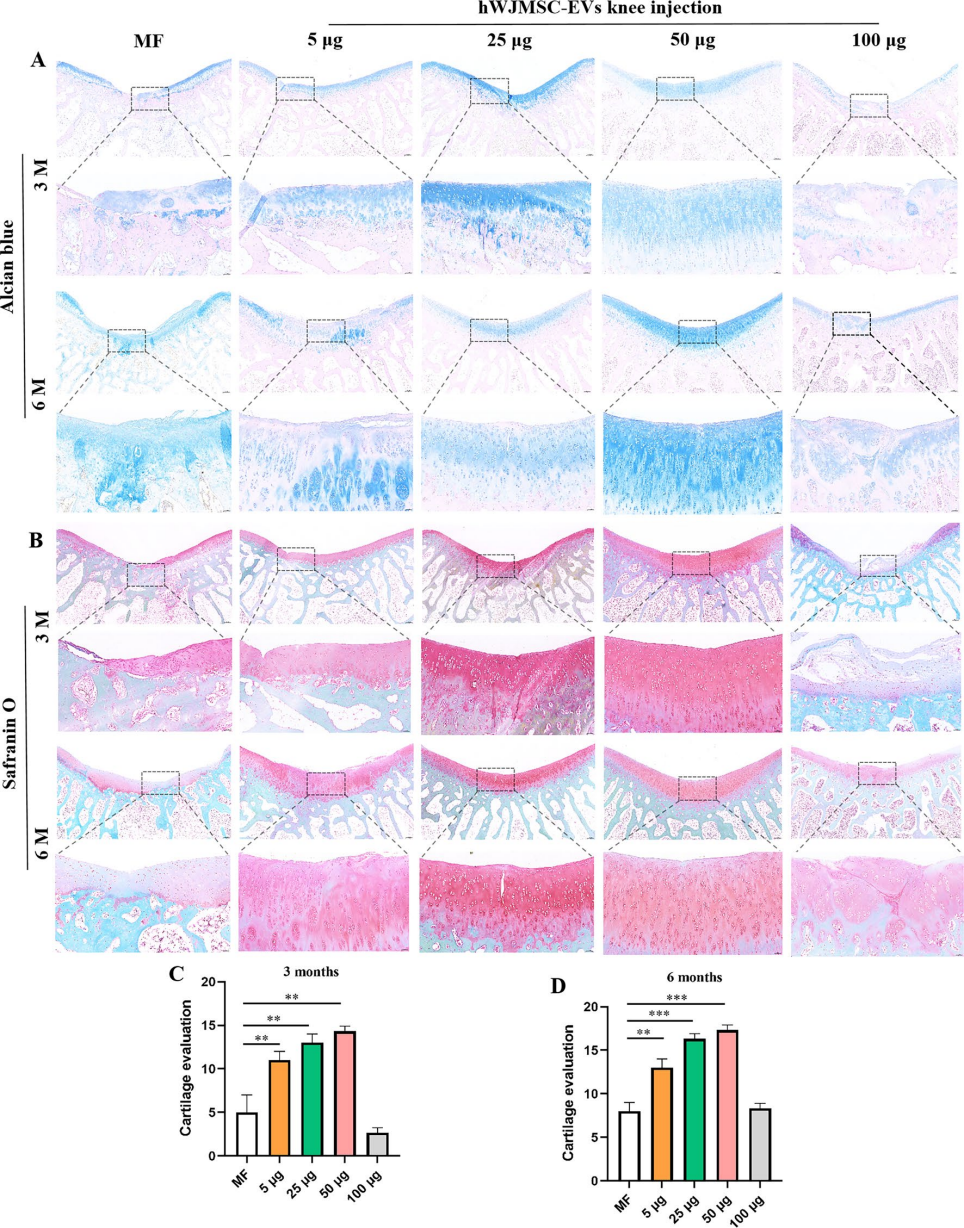
Alcian Blue and Safranin O staining. **A** Alcian Blue staining of regenerated cartilage. **B** Safranin O staining of regenerated cartilage. **C**, **D** Cartilage score, mean ± SD. Scale bar = 200 µm and 50 µm. *P < 0.05, **P < 0.01, **P < 0.005

Alcian Blue staining was used to analyze the regeneration of glycosaminoglycans (Fig. 9A). At 3 and 6 months postoperatively, the 50 µg/ml group had significantly better regeneration of glycosaminoglycans than the other groups. At 3 months postoperatively, there was a small amount of cartilage regeneration in the MF group, with continuous interruptions; the regenerated cartilage was a lighter blue color in the 5 and 100 µg/ml groups. At 6 months postoperatively, the MF group showed strong dark blue staining with a small amount of pale red cytoplasm. In the 5 µg/ml group at 6 months postoperatively, the Alcian Blue staining was interrupted and there was a small amount of fibrotic tissue in the middle. The regenerated cartilage in the 25 and 50 µg/ml groups showed the same staining as the surrounding normal cartilage at 6 months postoperatively. In the 100 µg/ml group, there was cartilage layer division with loose connections at 6 months postoperatively. No obvious fat cells were observed in any group.

Saffron O stains the polysaccharides in cartilage red (Fig. 9B). At 3 and 6 months postoperatively, the staining of regenerated cartilage was significantly better in the 25 and 50 µg/ml groups than the other groups. However, the 25 µg/ml group had subchondral bone healing at 3 months postoperatively, and had a central crack in the regenerated cartilage at 6 months. The polysaccharide content of regenerated cartilage in the MF group and the 5 and 100 µg/ml groups was significantly higher at 6 months postoperatively compared with that at 3 months postoperatively.

The histological scoring was consistent with the staining results. The cartilage score was significantly better in the 50 µg/ml group than the other groups at 3 and 6 months postoperatively (Fig. 9C, D).

#### Immunohistochemical evaluation of the distribution and changes in the expression levels of COL-I, COL-II, and COL-III

**Fig. 10:**
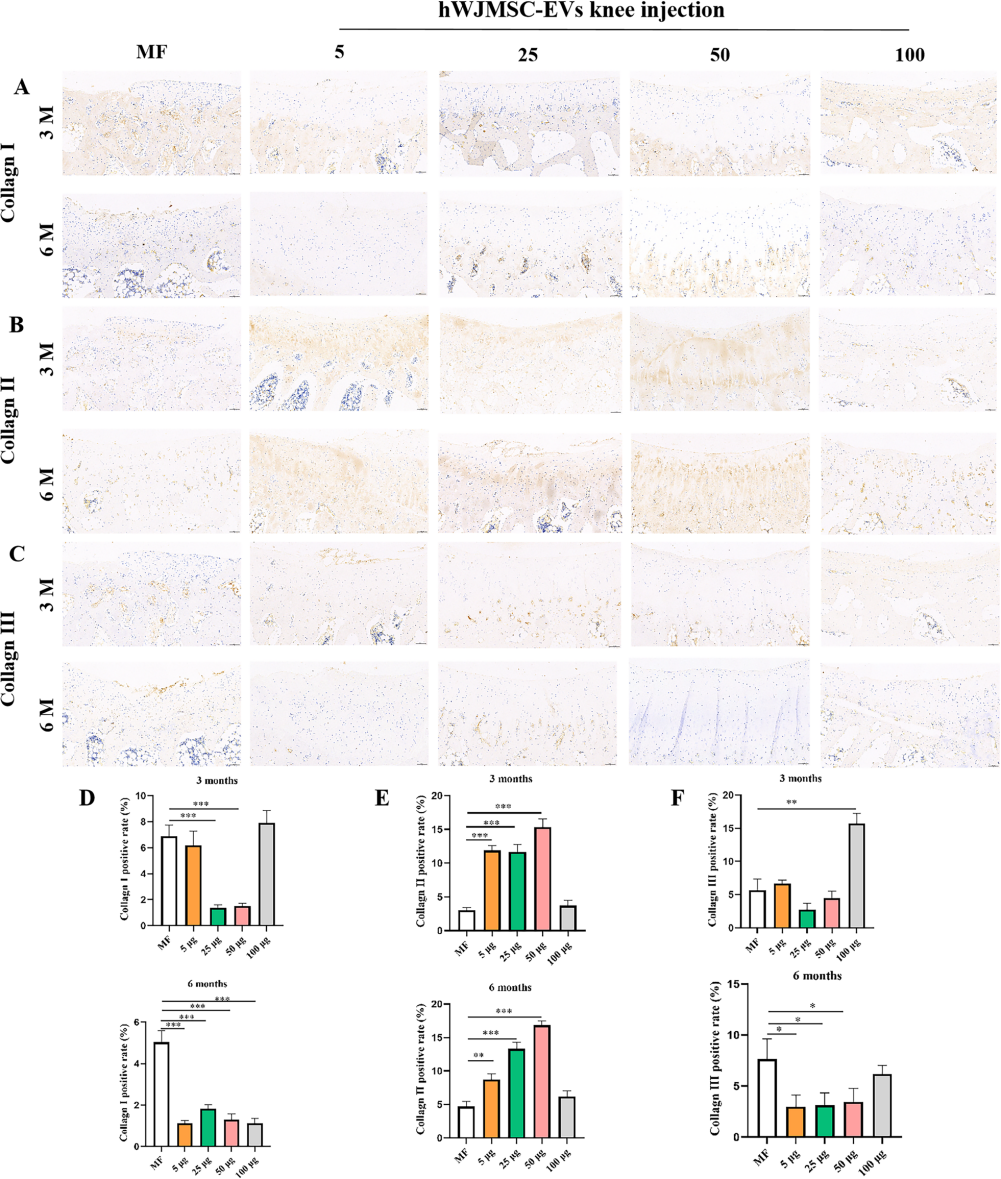
COL-I, COL-II, and COL-III content in regenerated cartilage. **A** COLL-I in regenerated cartilage. **B** COLL-II in regenerated cartilage. **C** COLL-III in regenerated cartilage. **D** Positive rate of COLL-I. **E** Positive rate of COLL-II. **F** Positive rate of COLL-III. Mean ± SD. Scale bar = 100 µm. *P < 0.05, **P < 0.01, **P < 0.005

We detected the expression levels of COL-I and COL-III related to fibrocartilage, which are indicated by brown staining. The 5 µg/ml group showed brown COL-I staining on the surface of regenerated cartilage at 3 months postoperatively, which had decreased at 6 months postoperatively. The 25 and 50 µg/ml groups expressed small amounts of COLL-I and COLL-III in the regenerated cartilage at 3 and 6 months postoperatively. At 3 months postoperatively, the MF group expressed a large amount of COL-I and a small amount of COL-III; as the regenerated cartilage healed, the amounts of COL-I and COL-III gradually increased. In the 100 µg/ml group, the regenerated cartilage expressed COLL-I and COLL-III at 3 months postoperatively, and showed improved expression of COLL-I and COLL-III at 6 months postoperatively (Fig. 10A, C). The quantitative results showed that the 100 µg/ml and MF groups had significantly greater expression levels of COLL-I and COLL-III at 3 and 6 months postoperatively compared with the other groups (Fig. 10D, F).

We further investigated the immunohistochemistry of COLL-II. The regenerated cartilage in the 5, 25, and 50 µg/ml groups expressed a large amount of COLL-II at 3 and 6 months postoperatively, while the MF and 100 µg/ml groups showed low expression levels of COLL-II in the regenerated cartilage (Fig. 10B). The quantitative results showed that the 50 µg/ml group had the highest content of COLL-II at 3 and 6 months postoperatively, and had a significantly greater COLL-II content than the MF group at both timepoints (Fig. 10E).

### Expression of collagen and mechanism in regenerated cartilage

**Fig. 11:**
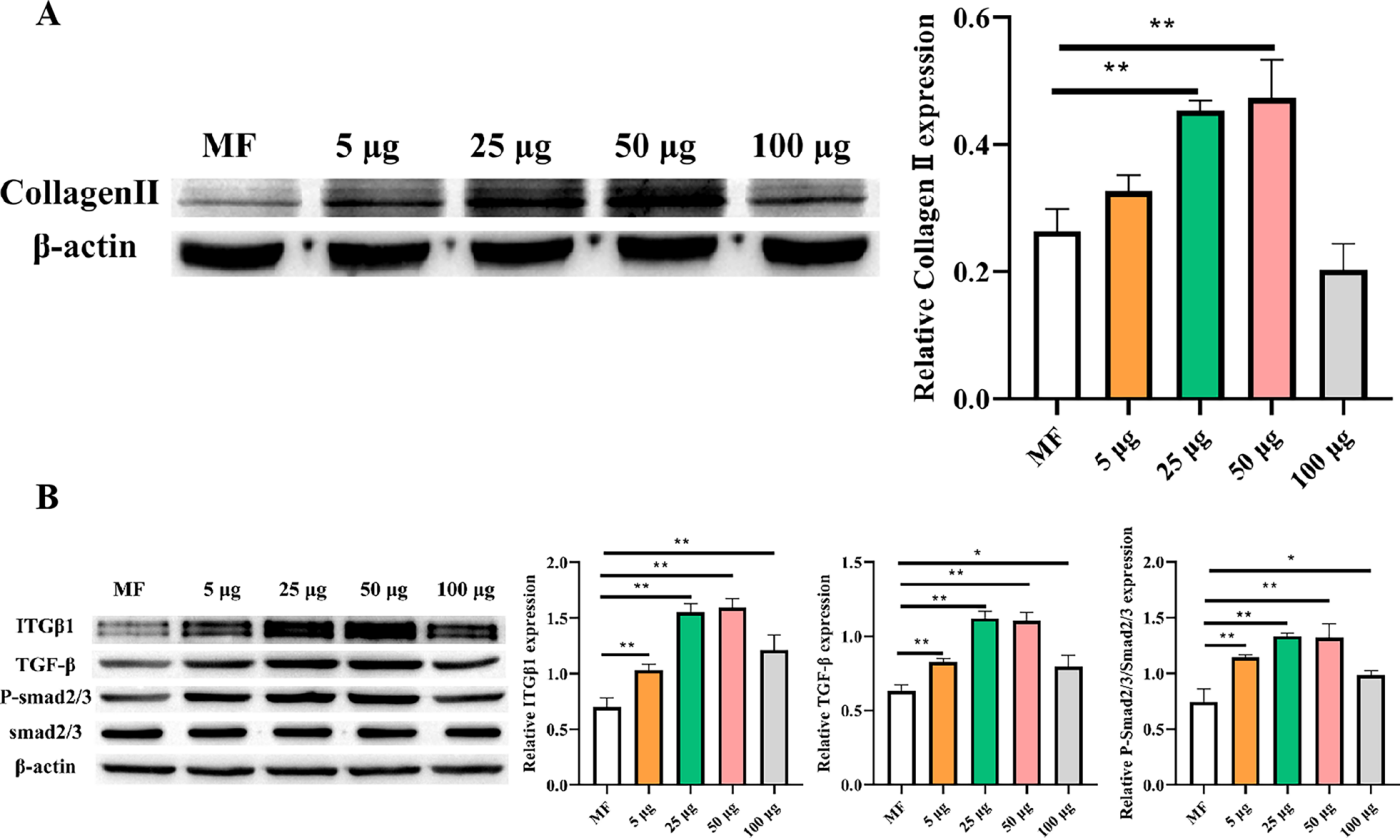
Western blot analysis to detect changes in COL-II, ITGB1, TGF-β, and Smad2/3 in regenerated cartilage. **A** Expression of COLL-II. **B** Expressions of ITGB1, TGF-β, and Smad2/3. Mean ± SD. *P < 0.05, **P < 0.01, **P < 0.005

We extracted the regenerated cartilage and detected the changes in COL-II, ITGB1, TGF-β, and Smad2/3 using Western blotting (Fig. 11A, B). The COL-II content in regenerated cartilage was significantly higher in the 50 µg/ml group than in the other groups (Fig. 11A). The changes in ITGB1, TGF-β, and Smad2/3 were directly proportional to the repair of regenerated cartilage (Fig. 11B), while the expression of Smad6 was inversely proportional to the repair situation (Additional file 1: Fig. S3); this indicates that intra-articular injection of HWJMSC-EVs upregulated the expressions of ITGB1, TGF-β, and Smad2/3, thereby promoting cartilage regeneration.

## Discussion

The present study was the first to show that HWJMSC-EVs combined with MF-stimulated BMSCs. Furthermore, EVs extracted from HWJMSCs promoted the vitality and proliferation of BMSCs and chondrocytes, induced BMSCs to differentiate into chondrocytes, and promoted the formation of glycosaminoglycans and COLL-II. In addition, after the injection of four different concentrations of EVs (5, 25, 50, and 100 µg/ml) into rabbit knee joints after MF surgery, micro-CT, histological staining, gross observation, ICRS scoring, immunohistochemical analysis of collagen content, and Western blotting protein detection showed that the 50 µg/ml concentration resulted in a significant improvement in cartilage repair compared with the other EV concentrations. Cellular and animal mechanism studies have shown that hWJMSC-EVs activate the TGF-β/Smad2/3 signaling pathway by increasing the expression of ITGB1 in BMSCs and chondrocytes.

Members of the ITGB superfamily interact with the ECM and cytoskeleton (cell–cell and cell–matrix) and play a crucial role in regulating cell proliferation and immune responses [36, 37]. As a member of the ITGB superfamily [38], ITGB1 plays a substantial role in regulating pathological skeletal damage [39, 40]. Our study found that HWJMSC-EVs increased the expression of ITGB1 in chondrocytes and hBMSCs. The key role of ITGB1 in cell migration, proliferation [41], and adhesion [42] has been reported, and the TGF-β/Smad2/3 pathway is known for its ability to inhibit cell apoptosis, promote tissue regeneration [43], and promote the proliferation and differentiation of MSCs and chondrocytes [44, 45]. In addition, the TGF-β/Smad2/3 pathway plays an important role in cartilage degeneration [46], maintenance of cartilage and the ECM [29, 47], and inhibition of the progression of osteoarthritis [48]. These functions are closely related to cartilage repair, suggesting that the ITGB1/TGF-β/Smad2/3 pathway is a promising endogenous protective signaling pathway that promotes cartilage regeneration. We found that the addition of HWJMSC-EVs and co-culture with BMSCs and chondrocytes significantly enhanced the vitality and proliferation ability of BMSCs and chondrocytes, mainly because of the high expressions of ITGB1 and TGF-β/Smad2/3. Furthermore, the addition of inhibitors of ITGB1 showed opposite results and significantly inhibited the expressions of TGF-β/Smad2/3, indicating that ITGB1 activated and inhibited the signaling pathways. Animal experiments in which we extracted regenerated cartilage for the detection of TGF-β/Smad2/3 at 3 months postoperatively confirmed the same findings as in the cell experiments. This phenomenon has not previously been reported in the process of cartilage repair.

Numerous studies have used EVs as substitutes for stem cells to repair articular cartilage defects. Compared with MSCs, EVs have advantages such as simple storage and transportation conditions, no tumorigenicity, no risk of disease transmission, and no risk of immune rejection [49, 50]. At present, the main exosomes derived for the treatment of cartilage injury include infrapatellar fat pad MSCs [51], synovial MSCs [52], human urine-derived stem cells [53], cartilage endplate stem cells [54], platelets [55], human anatomical MSCs [56], and Wharton’s jelly MSCs [57]. However, only HWJMSC-EVs are reported to contain the protein ITGB1, as confirmed in our experiments. Our study also confirmed that HWJMSC-EVs promoted the vitality, proliferation, and differentiation ability of BMSCs and chondrocytes. EVs from various sources have been confirmed to have the same function [58], but further research is needed to determine whether they have the same mechanism.

Endogenous BMSCs have the ability to self-renew, maintain knee joint stability, and repair damaged cartilage. In recent decades, endogenous BMSCs have been identified as a qualified source of cells for in vivo cartilage regeneration [59, 60]. In the MF technique, doctors drill holes into the subchondral bone to expel MSCs, cytokines, and platelets from the bone marrow, thereby stimulating cartilage regeneration. The MF technique is favored by most orthopedic doctors because of its minimal trauma, low technical requirements, high surgical safety, and fast postoperative recovery [7, 61]. However, because of the lack of factors that induce the differentiation of BMSCs into transparent cartilage and the fact that only a small number of BMSCs are recruited and exist normally, the formation of a large amount of fibrocartilage leads to poor long-term efficacy [59, 62]. The addition of HWJMSC-EVs after MF surgery substantially compensates for this disadvantage. It has been confirmed that TGF-β [63] and the multifunctional growth factor bone morphogenetic protein of the TGF-β superfamily play a crucial role in inducing and recruiting BMSCs [8]. However, there is currently no research on how HWJMSC-EVs activate TGF-β. We co-cultured HWJMSC-EVs with BMSCs and chondrocytes and performed in vitro experiments that demonstrated for the first time that the activation of TGF-β in cartilage regeneration is caused by the upregulation of ITGB1.

On the basis of the in vitro experiments, we combined hWJMSC-EVs with MF-stimulated BMSCs and studied the effects of the addition of different concentrations of EVs. The repair effect of 50 µg/ml of EVs was significantly better than the effect of the other four concentrations. The micro-CT results showed that the areas of subchondral bone defects in each group were connected to the edge of the normal subchondral bone at 3 months postoperatively; at 6 months postoperatively, there were more dense connections with a small central defect. These findings suggest that the regeneration of subchondral bone may gradually progress from the periphery to the center. Furthermore, the macroscopic observations showed that the density of connections in the subchondral bone defect area was directly proportional to the cartilage regeneration seen on micro-CT. This proves that the cartilage repair was closely related to the repair of the subchondral bone. However, the MF technique only induces the formation of fibrocartilage, with a low O’Driscoll history score [34] and poor long-term efficacy. In the present study, the addition of 50 µg/ml of EVs resolved this problem. This is consistent with a previous study that reported that EVs combined with hyaluronic acid improve the efficacy of cartilage repair in combination with the acellular cartilage matrix scaffold [64]. Another notable feature of transparent cartilage compared with fibrocartilage is its high content of COLL-II [65]. Immunohistochemical staining showed that the color was uniform in the 50 µg/ml group, indicating a high COLL-II content in the repaired tissue. The quantitative results also showed that the collagen content was significantly higher in the 50 µg/ml group than the other groups.

In the present study, the amount of cartilage repair did not increase with the increase in the concentration of EVs. When the EV concentration was 100 µg/ml, the regeneration of subchondral bone and cartilage was significantly inhibited. We believe that there are three main reasons for this phenomenon. Firstly, Zhang et al. [65] used 100 µg/ml EVs to significantly increase the proliferation, migration, and tubular formation ability of human venous endothelial cells in a rat model of full-thickness skin defects, thereby promoting vascular regeneration. Vascular regeneration has a significant inhibitory effect on the differentiation of MSCs into chondrocytes [66]. Secondly, EVs expose the pro-coagulation factor phosphatidylserine on the surface, which provides a catalytic surface for the formation of the coagulation complex of the coagulation cascade [67]. When EVs are injected after MF surgery, high concentrations of EVs accelerate the formation of blood clots and reduce the release and recruitment of BMSCs. Finally, our experiment only examined a portion of EVs, which contain numerous DNA, RNA, and proteins; the effective components and their functions in the joint microenvironment are not yet fully understood.

## Conclusion

The present results indicate that hWJMSC-EVs enhanced the vitality, proliferation, and differentiation ability of hBMSCs and chondrocytes. The injection of hWJMSC-EVs into the joint cavity at a concentration of 50 µg/ml significantly improved the bottleneck of transparent cartilage formation after MF surgery. This was mainly because hWJMSC-EVs increased the expression of ITGB1 to activate the TGF-β/Smad2/3 signaling pathway. This method of hWJMSC-EVs injection may provide a simple solution for MF-mediated cartilage regeneration, further promoting the widespread application of enhanced MF treatment.

### Supplementary Information

**Additional file 1: Figure S1.** HWJMSCs and HWJMSC-EVs upregulate Smad6 in hBMSCs and chondrocytes. **Figure S2.** HWJMSC-EVs promote the expression of ITGB1 in BMSCs and chondrocytes. **Figure S3.** Western blot was used to detect regenerated cartilage.

## Data Availability

The datasets generated during and/or analyzed during the current study are available from the corresponding author on reasonable request.
