# Supplementary material for: HWJMSC-EVs promote cartilage regeneration and repair via the ITGB1/TGF-β/Smad2/3 axis mediated by microfractures
Source: J Nanobiotechnology. 2024 Apr 12;22:177. doi: 10.1186/s12951-024-02451-2 (PMC11015550; doi:10.1186/s12951-024-02451-2)
Supplement: Supplementary file 1 — Additional file 1: Figure S1. HWJMSCs and HWJMSC-EVs upregulate Smad6 in hBMSCs and chondrocytes. Figure S2. HWJMSC-EVs promote the expression of ITGB1 in BMSCs and chondrocytes. Figure S3. Western blot was used to detect regenerated cartilage. [file 12951_2024_2451_MOESM1_ESM.docx]

**HWJMSC-EVs promote cartilage regeneration and repair via the ITGB1/TGF-β/Smad2/3 axis mediated by microfractures**

Zhian Chen^a,c^,Tianhua Zhou^b^,Huan Luo^a^ ,Zhen Wang^a^,Qiang Wang^c^,Rongmao Shi^b^,Zian Li^c^,Rongqing Pang^c#^, Hongbo Tan^b#^

^a^Graduate School, Kunming Medical University, Kunming City, Yunnan Province, China

^b^Department of Orthopaedics, People’s Liberation Army Joint Logistic Support Force 920th Hospital, Kunming City, Yunnan Province, China

^c^Basic Medical Laboratory, People’s Liberation Army Joint Logistic Support Force 920th Hospital, Kunming City, Yunnan Province, China

**^#^Correspondding author:**Rongqing Pang and Hongbo Tan, People’s Liberation Army Joint Logistic Support Force 920th Hospital, Kunming City, Yunnan Province, China


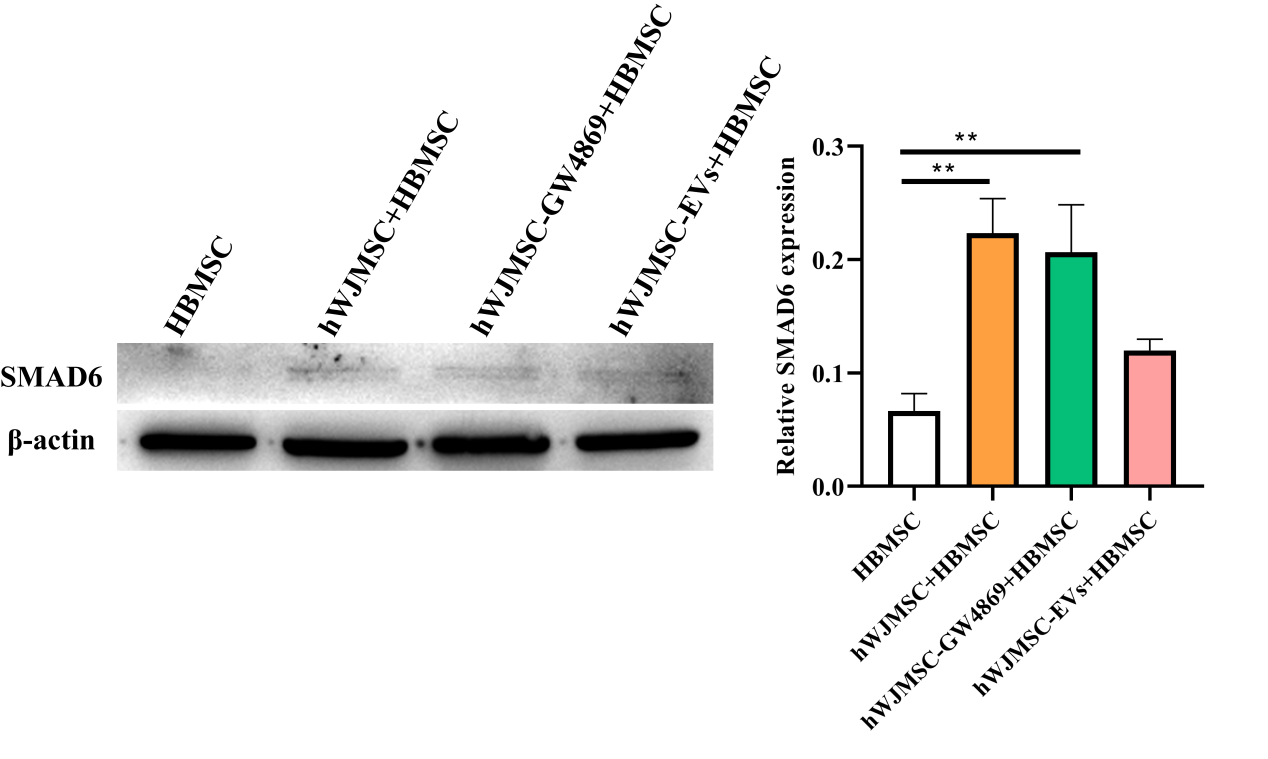


Figure S1.The expressions of Smad6 in hBMSCs detected by Western blotting. Mean ± SD, n = 3. **P* < 0.05, ***P* < 0.01.


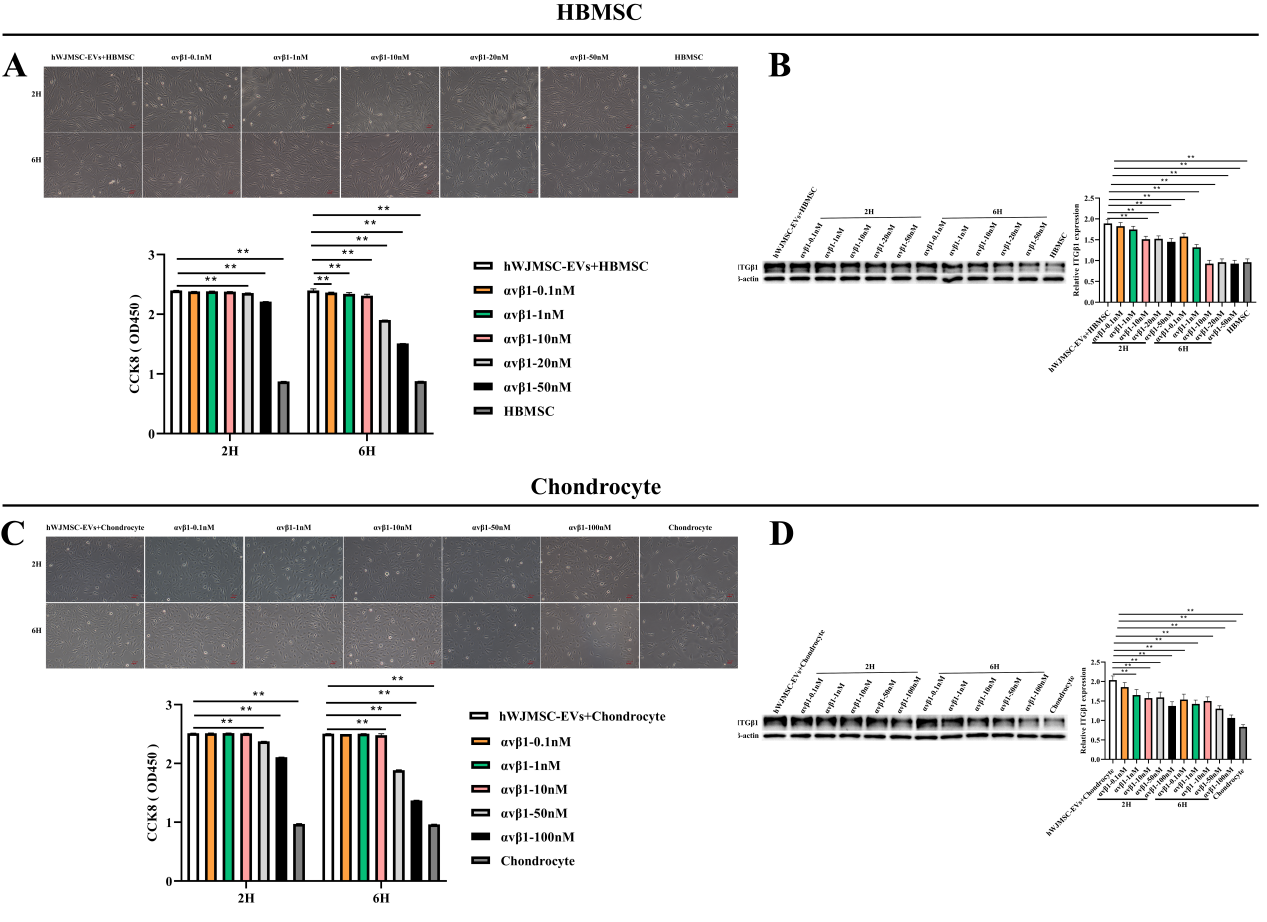


Figure S2.HWJMSC-EVs promoted the expression of ITGB1 in BMSCs and chondrocytes. (A and C) The viability of treated hBMSCs and chondrocytes was detected by CCK8 assay and observed under light microscopy. Scale bar = 50 µm. Mean ± SD, n = 3. **P* < 0.05, ***P* < 0.01. (B, D,) The expressions of ITGB1 in cells (hBMSCs and chondrocytes) detected by Western blotting. Mean ± SD, n = 3. **P* < 0.05, ***P* < 0.01.


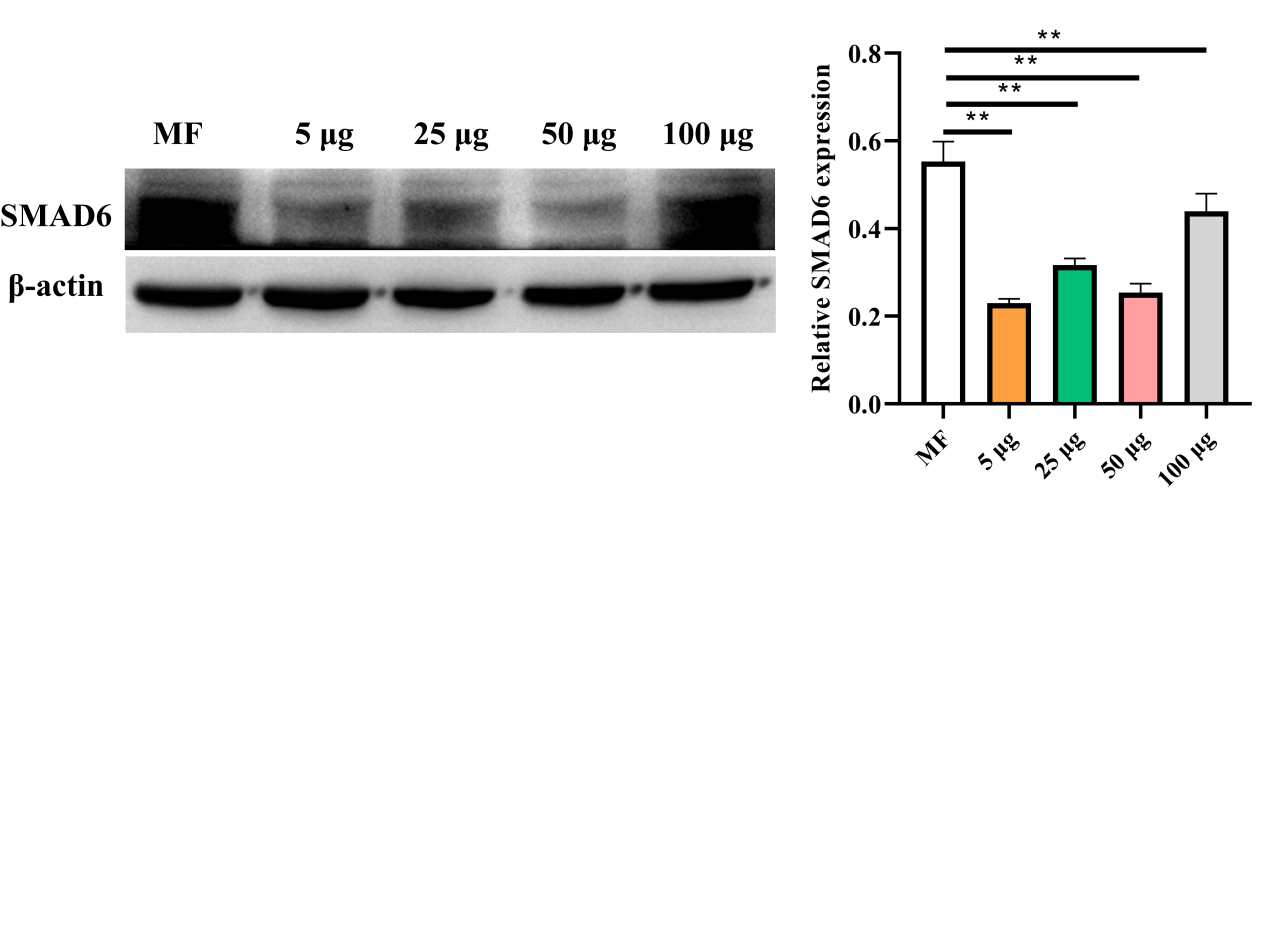


Figure S3.Western blot analysis to detect changes in Smad6 in regenerated cartilage.Mean ± SD. **P* < 0.05, ***P* < 0.01,***P* < 0.005.
